# Efficacy of cannabis oil on appetite and quality of life in systemic sclerosis patients: a randomized placebo-controlled trial

**DOI:** 10.1186/s42238-025-00342-3

**Published:** 2025-10-24

**Authors:** Veeradej Pisprasert, Bungon Sripanichkulchai, Teerawat Khannongpho, Amonrat Jumnainsong, Ajanee Mahakkanukrauh, Siraphop Suwannaroj, Patnarin Pongkulkiat, Tippawan Onchan, Somdej Kanokmedhakul, Apichart So-ngern, Chingching Foocharoen

**Affiliations:** 1 Department of Medicine, Faculty of Medicine, Khon Kaen University, Khon Kaen, 40002 Thailand; 2 Faculty of Pharmaceutical Sciences, Khon Kaen University, Khon Kaen, 40002 Thailand; 3 Department of Nutrition, Srinagarind Hospital, Khon Kaen University, Khon Kaen, 40002 Thailand; 4 Faculty of Associated Medical Sciences, Khon Kaen University, Khon Kaen, 40002 Thailand; 5 Department of Chemistry, Faculty of Science, Khon Kaen University, Khon Kaen, 40002 Thailand

**Keywords:** Scleroderma and related disorders, Quality of life, Clinical trials and methods, Inflammation, Interventional study

## Abstract

**Background:**

The efficacy of cannabinoids as appetite stimulants in chronic wasting disorders is well established; however, their role in systemic sclerosis (SSc) remains to be elucidated. We aimed to evaluate the efficacy of cannabis oil on appetite, inflammatory markers, quality of life (QoL), and adverse events in patients with SSc compared to placebo.

**Methods:**

A randomized placebo-controlled trial was conducted in 27 SSc patients with anorexia or malnutrition, according to sample size analysis. Patients with overlap connective tissue diseases, malignancies, or severe medical conditions were excluded. Participants were randomized 1:1 to receive either cannabis oil or placebo (two drops sublingual twice daily). The endpoints included changes in appetite grading using the visual analogue scale (VAS), body weight (BW), daily calorie intake, inflammatory markers, and QoL assessed using the EuroQol-5 Dimension (EQ-5D).

**Results:**

Thirteen patients in each group completed the study (66.7% were female, and 77.9% had diffuse cutaneous SSc). The cannabinoid group trended toward greater improvements in appetite, satisfaction with eating, ability to eat more, BW, daily calorie intake, health VAS, and reduced inflammatory markers than the placebo group, although the differences were not statistically significant. Transferrin, transforming growth factor-β, and serum albumin levels did not differ between the groups. The VAS score for hunger significantly increased in the treatment group (*p* < 0.001) but not in the placebo group. One patient in the treatment group developed severe hyponatremia and was withdrawn from the study.

**Conclusion:**

Cannabis oil showed a trend toward improving appetite, BW, calorie intake, and QoL in SSc patients with anorexia, though most results were not statistically significant. Hunger VAS scores increased significantly, and inflammatory markers showed some reduction. Larger studies are needed to confirm these findings.

**Clinical trial number:**

NCT05416697, registered on June 9, 2022.

**Supplementary Information:**

The online version contains supplementary material available at 10.1186/s42238-025-00342-3.

## Key messages

Cannabis oil can improve appetite and have an anti-inflammatory action but the effects in systemic sclerosis (SSc) are limited.

Appetite stimulating, anti-inflammatory, and antifibrotics of cannabinoids effect of cannabis oil are poorly understood.

**Supplementary Information:**

The online version contains supplementary material available at 10.1186/s42238-025-00342-3.

## Introduction

Historical references to the beneficial effects of cannabis can be traced back 4,000 years. Cannabinoids are usually referred to as tetrahydrocannabinol (THC) or cannabidiol (CBD). THC is a prominent, psychoactive cannabinoid. It has neuropsychiatric and antiemetic effects, so it is approved for use as an antiemetic for nausea and vomiting associated with chemotherapy, analgesic for neuropathic pain or cancer pain, and appetite stimulant for chronic wasting disease. Brisbois et al. (2011) found that THC in dose 2.5 mg twice daily had significant greater effect on food taste, quality of life and sleep quality than placebo treatment in cancer patients. In contrast, it can also cause an unsatisfactory psychoactive effect (May and Glode 2016). The appetite stimulation by THC was demonstrated via the cannabinoid receptor 1 (CB1) receptor in the hypothalamus, and the mesolimbic reward system then regulated the eating motivation (Vadivelu et al. 2018). In contrast, it can also cause an unsatisfactory psychoactive effect (May and Glode 2016). The appetite stimulation by THC was demonstrated via the CB1 receptor in the hypothalamus, and the mesolimbic reward system then regulated the eating motivation (Vadivelu et al. 2018). 

CBD is a cannabinoid compound that has an anti-inflammatory effect but no psychoactive action (Nagarkatti et al. 2009). CBD primarily binds to CB1 receptor, which is mainly present on neurons of the basal ganglia and hippocampus, and cannabinoid receptor 2 (CB2), which is principally located in the immune system tissue and cells (Zurier and Burstein 2016). CBD has an anti-inflammatory action via the non-cyclooxygenase pathway, so the action and side effects of CBD are similar to those of non-steroidal anti-inflammatory agents (NSAIDs). However, the mechanism underlying the anti-inflammatory effects of CBD remains unclear.

Cannabinoids also influence the immune system and are involved in fibrogenesis. Servettaz et al. (2010) reported the effects of cannabinoid agonists, particularly selective CB2 receptor agonists, on the development of lung and skin fibrosis in SSc-induced mice. They also found a role for CB2 receptors in the development of SSc in an animal model. The study found that SSc-induced mice had less skin thickness, less collagen accumulation, and a more significant reduction in skin fibroblast proliferation rate after CB2 receptor agonist injection than untreated mice (Servettaz et al. 2010). The CB2 receptor agonist also decreased collagen type I concentration in the lungs of the treated mice compared to the untreated mice. In addition, both non-selective cannabinoid agonists and CB2 agonists influenced B cells by preventing the development of autoantibodies after exposure to hypochlorous acid, an agent that can induce SSc, compared to untreated mice. The results concluded that cannabinoid agonists, particularly CB2 receptor agonists, could prevent skin and lung fibrosis and autoantibody production in SSc-induced mice. To date, there has been no study on the effect of cannabinoids on the long-term outcomes of skin fibrosis in patients with SSc.

SSc is a connective tissue disease in which skin tightness is the hallmark. The earliest clinical features of SSc typically include Raynaud’s phenomenon and puffy hands or feet. However, a significant gap in clinical management exists, as there are no consensus-driven, evidence-based guidelines for either the early diagnosis of the disease or for strategies to prevent its progression to an established state (Smith et al. 2018). The disease is classified into two significant subsets: limited cutaneous systemic sclerosis (lcSSc) and diffuse cutaneous systemic sclerosis (dcSSc), depending on the extent of skin tightness (Silver 1991). Malnutrition and/or weight loss is a complication in SSc (Harrison et al. 2012). The complications are possibly related to gastrointestinal involvement, inflammation, immunosuppressant agents, or mood disturbances, which can affect food appetite or eating behavior (Harrison et al. 2012; Baron et al. 2009). Cannabinoid is an agent that affects appetite, as mentioned above; hence, it would improve appetite in SSc patients.

Transforming growth factor-β (TGF-β) is a major cytokine that plays a key role in SSc pathogenesis. TGF-β is produced by T-cells, monocytes, and platelets. TGF-β regulates fibroblast differentiation and proliferation (Balbir-Gurman and Braun-Moscovici 2012). According to a previous study, CBD, particularly the CB2 receptor agonist, can reduce skin and lung fibrosis in SSc-induced mice, and TGF-β should also be affected. However, there are no studies on the effects of cannabinoids on TGF-β levels in SSc-induced mice or SSc patients.

Evidence of the effect of cannabinoids on appetite stimulation in SSc is limited; therefore, we aimed to investigate the efficacy of cannabis oil on appetite, quality of life, and key cytokine levels in SSc compared with placebo, as well as to document any adverse events associated with cannabinoids in these patients.

## Methods

This randomized placebo-controlled study included SSc patients aged between 18 and 70 years who: were diagnosed according to the American College of Rheumatology (ACR)/the European Alliance of Associations for Rheumatology (EULAR) 2013 classification criteria; had anorexia or malnutrition status; did not receive steroid equivalent to prednisolone dose more than 10 mg/d; received a stable dose of steroid, immunosuppressant and/or vitamin or its supplement within 2 weeks before enrollment; stopped anxiolytics, hypnotics, or sleeping pills at least 2 weeks before enrollment; and, understood and were able to read and write Thai. We excluded the following patients who: (a) had overlap with other connective tissue diseases; (b) were pregnancy or lactation; (c) had bedridden and confined to no self-care; (d) had evidence of active malignant disease; (e) presented of uncontrolled or severe medical problems including diabetes mellitus, asthma, angina, cardiovascular, thyroid, hepatic or renal diseases (serum creatinine > 1.4 mg/dL); (f) presented of active infection that needs systemic antibiotic; (g) had previous allergy of cannabinoid or its derivatives; (h) received concomitant illegal drug used (amphetamine or its derivative, cocaine); (i) had history of previous cannabinoid using or concomitant any herbal included cannabinoid used; (j) were on anxiolytics, hypnotics or sleeping pills used; k) were in a period that needs immunosuppressant dose adjustment; l) had active SSc that needs closed monitoring for disease progression (pulmonary hypertension, proteinuria, microscopic hematuria, digital gangrene, and progressive interstitial lung disease); m) had unstable cardio-pulmonary disease (angina, peripheral vascular disease, cerebrovascular disease and arrhythmia) and risk of cardiovascular disease; n) had history of schizophrenia, concurrent active mood disorder, or anxiety disorders; and, o) received the following medications that cause drug interaction with cannabinoid: fluoxetine, rifampicin, carbamazepine, warfarin, clobazam, and fluoroquinolones. Recruitment for the study started in November 2022 and continued until July 2024.

### Intervention

All eligible subjects were randomly assigned to either cannabis oil or placebo in a 1:1 ratio by computer generation with a block of 4. For the treatment group, the patients received cannabis 2.7 mg THC-2.5 mg CBD/mL 1 drop (0.73 mg THC and 0.81 mg CBD) sublingual twice daily at baseline for 1 week, then titrated up to 2 drops twice daily (2.92 mg THC and 3.24 CBD per day) if tolerated and continued with the dose until the end of the study (totally 71.54 mg THC and 79.38 mg CBD/patients/study period). The other group received placebo 1 droplet twice daily at baseline for 1 week and then titrated up to 2 drops twice daily and continued until the completion of the study. As a standard therapy for underlying SSc, physicians can adjust concomitant medication apart from prohibited medication.

### Drug information

Cannabis oil extract, stored in a light brown bottle, comprises both THC and CBD in a 1:1 ratio (27 mg of THC plus 30 mg of CBD/g extraction or 27 mg of THC plus 30 mg of CBD/mL). One drop of cannabis oil contained 0.73 mg THC and 0.81 mg of CBD (37 drops/mL), and one bottle of cannabis oil contained 8 g of cannabis extract, which was stored at 4 °C. It was a product of the Faculty of Pharmaceutical and Science project funded by Khon Kaen University.

Cannabis was extracted from the stems, leaves, and flowers of Cannabis sativa. The resource was obtained from the Office of the Narcotics Control Board, Government Pharmaceutical Organization, and Faculty of Agriculture, Khon Kaen University. It is a controlled substance according to the standards of the Thai Herbal Pharmacopoeia.

#### CBD Preparation process

Dry cannabis was ground thoroughly and fermented in 95.00% alcohol for 1 d at a 1:5 ratio. The fluid was then separated from the cannabis and fermented again. The fermented cannabis was then evaporated in water with dry evaporators, the yield was calculated, and the proportions of THC and CBD analyzed. The extracted product was run in either column chromatography or solvent to obtain the THC-enriched and CBD-enriched fractions. Before use as an experimental study drug, the cannabis extract was evaluated for heavy metal and pesticide contamination which had to contain less than 4 ppm arsenic, less than 0.3 ppm cadmium, less than 10 ppm lead, less than 0.5 ppm mercury, less than 5 × 10^4^ CFU/g total yeast and molds count, less than 5 × 10^5^ CFU/g total aerobic microbial count, no *Escherichia coli*, *Staphylococcus spp*, *Salmonella spp.* Or *Clostridium spp.*, and no pesticides, chlorine, phosphate, carbamate, or pyrethroid over the Maximum Residue Level of 0.05 ppm.

### Data collection

Baseline assessment included medical history, demographic data, clinical characteristics of SSc, food appetite grading by the questionnaire on appetite, and desire for specific food types comprising 8 questions; each question was graded using a visual analog scale (VAS) range from 0–10 (Flint et al. 2000), quantity and calorie of food intake estimated by dietary history, quality of life evaluated by EuroQol-5 Dimension (EQ-5D) scoring, routine laboratory assessment in SSc (complete blood count, erythrocyte sedimentation rate (ESR), C-reactive protein (CRP), renal function, liver function, urinalysis, creatine kinase, thyroid function test), chest radiography, pulmonary function test, echocardiography (chest radiography, pulmonary function test, and echocardiography are allowed to use the previous data of within 3 months before enrollment), blood test for cytokine (TGFβ) levels, and transferrin level. At week 2, after receiving the study drug or placebo, the patients were contacted by telephone to determine the health status and side effects of the study drug. At week 4 (study endpoint), data were collected and compared with the baseline assessment. All patients had a follow-up visit to evaluate their health status and safety profile 2 weeks after cessation of the study medication (week 6). In case of serious adverse events related to the study drug, patients were required to have a follow-up visit to evaluate the safety profile after cessation of the study medication.

The study endpoints included changes in appetite evaluated using the VAS, changes in food intake quantity evaluated by dietary history (calculated as daily calorie intake per kilogram (kg) of body weight), changes in serum transferrin levels, changes in quality of life evaluated by EQ-5D, change in key cytokine levels (TGF-β) compared to baseline, comparison between the treatment and placebo groups, and adverse drug reactions or events.

We planned to terminate the study if the prevalence of serious adverse events was more than 10–15% or more than 30% of subjects undergoing an exacerbation of disease requiring further investigation and/or changes in background therapy, such as coughing, tiredness, and increased acid reflux.

**Fig. 1:**
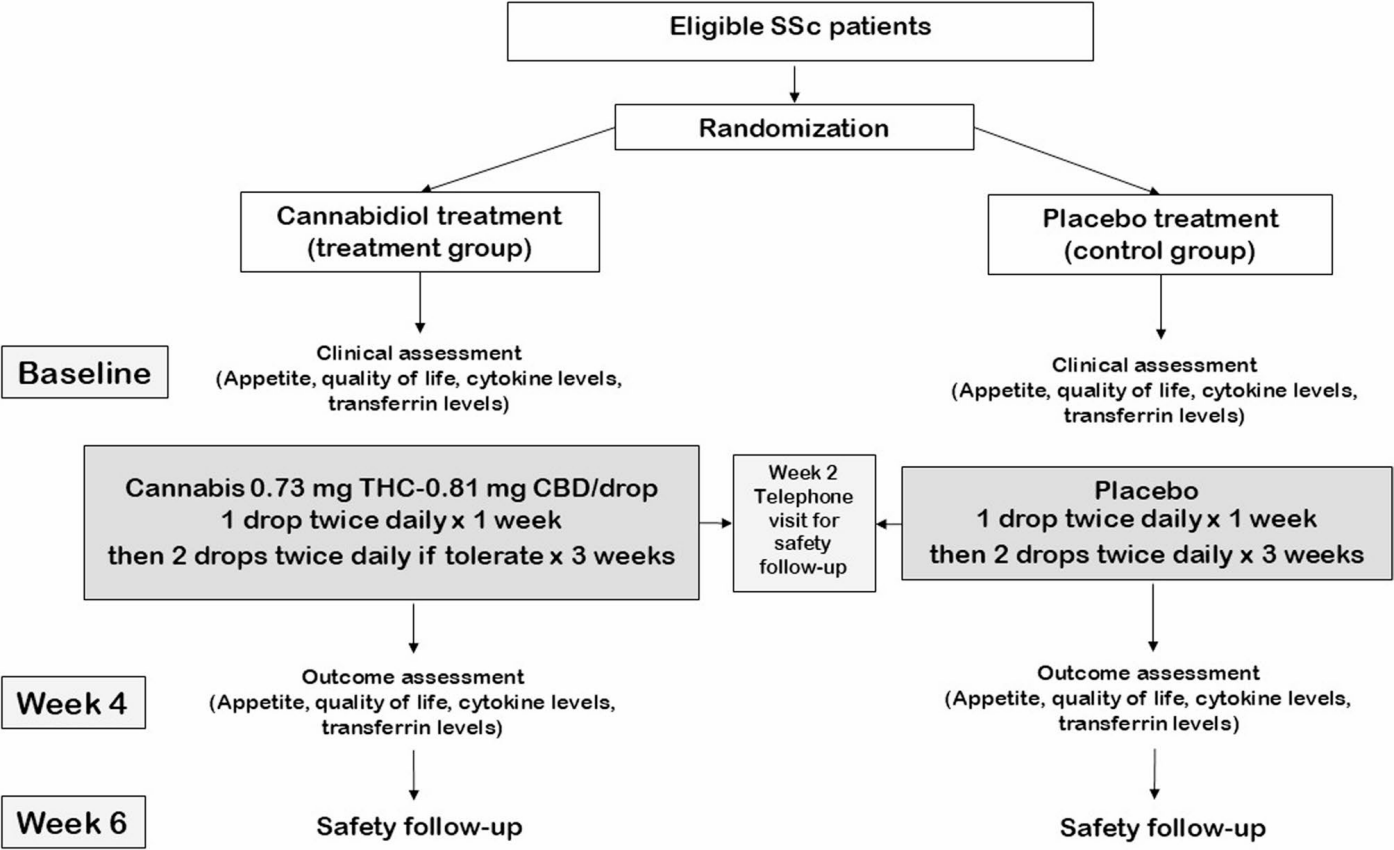
Study flow

The study flow is presented in Fig. 1.

### Operational definitions

The diagnosis of SSc was based on the 2013 ACR/EULAR Classification Criteria for Scleroderma (Hoogen et al. 2013). SSc was classified as the limited or diffuse type according to the classification of LeRoy et al. (1988) lcSSc is characterized by skin tightness of the face, hands, feet, forearms, and legs, whereas dcSSc is characterized by skin tightness of the trunk and both extremities.

Anorexia was defined if the patient complained self-reported of a decreased appetite with VAS for appetite ≤ 70 (Blauwhoff-Buskermolen et al. 2016). Malnutrition was defined according to subject global assessment class B or C (weight loss at least 5% within 6 months with no subsequent gain, in conjunction with subcutaneous fat or muscle loss, a reduction in dietary intake, and/or body mass index (BMI) ≤ 18.49 Kg/m^2^) (Duerksen et al. 2021). 

Serious adverse events associated with the study drug included symptoms that cause discomfort, disturb daily life, lead to life-threatening events (i.e., psychosis, severe cardiovascular events, signs of vascular insufficiency [ischemic ulcer or gangrene]), or needs hospitalization due to any causes or related to study medication.

### Statistical analysis

Patient baseline characteristics were summarized using descriptive statistics. Continuous variables were presented as means and standard deviations or medians and interquartile ranges, as appropriate. Categorical variables were summarized as frequencies and percentages. Analyses were performed using Student’s unpaired t-test or Mann-Whitney U test to evaluate differences in continuous outcomes between the treatment and placebo groups, depending on data distribution. Categorical outcomes were compared using the chi-square test or Fisher’s exact test, as appropriate, to evaluate differences between the treatment and placebo groups. To compare outcomes between baseline and week 4 after treatment, the paired Student’s t-test was used for continuous data, and McNemar’s test was applied to categorical data.

For handling missing data, patterns of randomness were assessed. Subgroup and sensitivity analyses were not conducted to explore treatment effects across different demographic groups due to the low sample size. Adverse events were reported descriptively. All p-values were two-tailed, and statistical significance was set at *p* < 0.05. All statistical analyses were performed using STATA version 16.0 (Stata Corp., College Station, TX, USA).

### Sample size calculation

The sample size was determined based on detecting a statistically significant difference in the primary outcome, the mean appetite VAS score, between the treatment and placebo groups among patients with cancer (Brisbois et al. 2011). According to the literature, the expected mean (standard deviation, SD) appetite score was 60.6 (11.3) in the treatment group and 50.9 (10.7) in the placebo group. The clinically meaningful difference between the two groups was set at 9.7. Assuming a two-tailed significance level of 0.05 and a power of 0.80, the sample size for each group was calculated for comparing two independent means. A minimum of 11 participants per group was needed to detect a difference with the desired power and significance level. Considering the potential dropout rate of 20%, the sample size was adjusted to 14 participants per group.

## Results

**Fig. 2:**
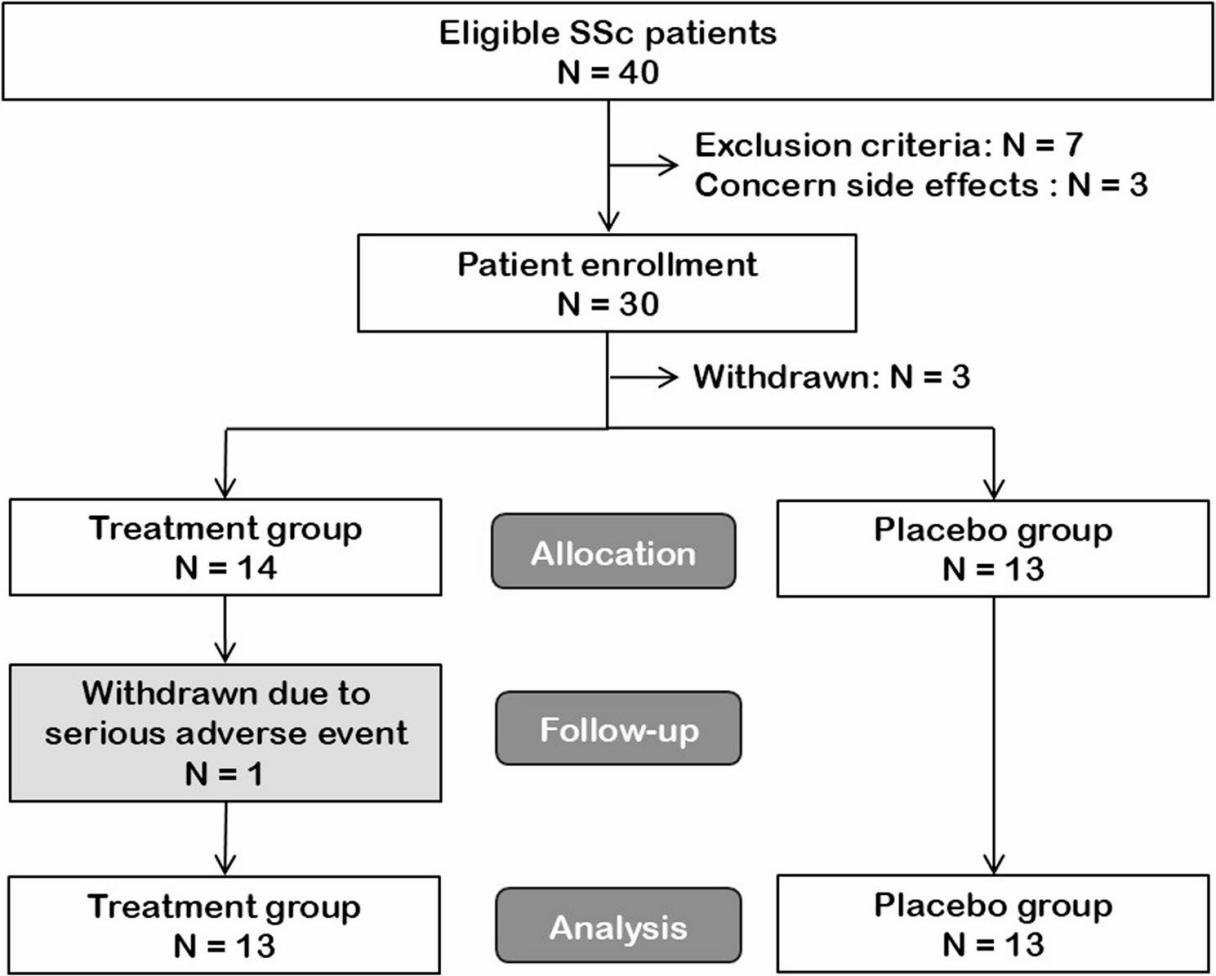
CONSORT diagram of patient flow

A total of 40 eligible patients were initially identified, but 10 were excluded because they met the exclusion criteria (7 cases) or because of concerns about the side effects of cannabinoids (3 cases). The remaining 30 patients were included in the study. Subsequently, three patients withdrew, leaving 27 included and randomized participants: 14 in the treatment group and 13 in the placebo group. One patient in the treatment group experienced serious adverse events that required hospitalization and was withdrawn from the study, resulting in 26 cases (13 in each group) for the analysis. The CONSORT diagram of patient flow is presented in Fig. 2, and the CONSORT checklists are included as Supplementary File 1.

Of the 27 allocated patients, most were female (9 in each group) and had the dcSSc subset (10 in the treatment group and 11 in the placebo group). The overall mean age was 55.9 years (SD 10.4), and the mean disease duration was 7.8 years (SD 6.1). Baseline clinical characteristics were similar between the treatment and placebo groups, except for transferrin levels and daily calorie intake per kg body weight, which were higher in the treatment group than in the placebo group (*p* = 0.01 and 0.02, respectively). The baseline clinical characteristics of the patients are presented in Table 1.

**Table 1:** Baseline clinical characteristics

Clinical characteristics	All cases *N* = 27	Treatment group *N* = 14	Placebo group *N* = 13	*p*-value^a^
Age at onset (years), mean (SD)	48.1 (13.0)	50.8 (9.8)	45.1 (15.6)	0.26
Age on baseline visit (years), mean (SD)	55.9 (10.4)	57.6 (9.0)	54.0 (11.7)	0.38
Duration of disease (years), mean (SD)	7.8 (6.1)	6.8 (3.7)	8.9 (7.9)	0.37
Female, *n* (%)	18 (66.7)	9 (64.3)	9 (69.2)	0.99
Diffuse cutaneous SSc subset, *n* (%)	21 (77.9)	10 (71.4)	11 (84.6)	0.65
Body mass index (kg/m^2^), mean (SD)	17.9 (1.9)	17.8 (2.1)	18.1 (1.6)	0.75
SSc clinical characteristics				
WHO functional class				0.57
I, *n* (%)	14 (51.9)	8 (57.1)	6 (46.2)	
II, *n* (%)	13 (48.2)	6 (42.9)	7 (53.9)	
Raynaud’s phenomenon, *n* (%)	14 (51.9)	7 (50.0)	7 (53.9)	0.84
Digital ulcer, *n* (%)	5 (18.5)	4 (28.6)	1 (7.7)	0.34
Telangiectasia, *n* (%)	17 (63.0)	9 (64.3)	8 (61.5)	0.99
Salt and pepper skin appearance, *n* (%)	19 (70.4)	11 (78.6)	8 (61.5)	0.42
Edematous skin, *n* (%)	2 (7.4)	0	2 (15.4)	0.22
Tendon friction rub, *n* (%)	4 (14.8)	2 (14.3)	2 (15.4)	0.99
Hand deformity, *n* (%)	17 (63.0)	8 (57.1)	9 (69.2)	0.70
Synovitis, *n* (%)	0	0	0	NA
Dysphagia, *n* (%)	11 (40.7)	3 (21.4)	8 (61.5)	0.054
Heartburn, *n* (%)	5 (18.5)	3 (21.4)	1 (15.4)	0.99
Stomach involvement, *n* (%)	5 (18.5)	2 (14.3)	3 (23.1)	0.65
Intestinal involvement, *n* (%)	5 (18.5)	1 (7.1)	4 (30.8)	0.17
Interstitial lung disease, *n* (%)	19 (70.4)	9 (64.3)	10 (76.9)	0.68
Pulmonary hypertension, *n* (%)	1 (3.7)	0	1 (7.7)	0.48
mRSS (points), median (IQR)	9 (2–19)	11.5 (2–19)	6 (2–23)	0.95
Laboratory tests				
Hb (g/dL), mean (SD)	12.0 (1.7)	12.2 (2.0)	11.8 (1.4)	0.58
Serum albumin (g/dL), mean (SD)	3.9 (0.4)	4.0 (0.4)	3.8 (0.4)	0.17
ESR (mm/h), mean (SD)	72 (30.5)	70 (28.0)	74 (34.4)	0.77
CRP (mg/dL), median (IQR)	3.0 (1.1–6.3)	3.2 (1.1–10.8)	2.4 (1.8–5.6)	0.57
Transferrin levels (g/L), mean (SD)	2.1 (0.4)	2.3 (0.4)	1.9 (0.4)	0.01*
TGFβ (pg/mL), mean (SD)	5.9 (2.4)	5.7 (2.4)	6.1 (2.3)	0.67
Treatment				
Prednisolone (mg/d), median (IQR)	3.8 (0–10)	1.9 (0–5)	5 (0–10)	0.31
Cyclophosphamide, *n* (%)	2 (7.4)	1 (7.1)	1 (7.7)	> 0.99
Mycophenolate, *n* (%)	8 (29.6)	3 (21.4)	5 (38.5)	0.33
Methotrexate, *n* (%)	5 (18.5)	2 (14.3)	3 (23.1)	0.56
VAS for self-reported				
Hunger (points), mean (SD)	5.9 (2.3)	6.2 (1.4)	5.5 (3.0)	0.46
Satisfaction in eating (points), mean (SD)	5.4 (2.1)	5.4 (2.3)	5.5 (1.9)	0.83
Feeling of fullness (points), mean (SD)	7.6 (1.8)	8 (1.6)	7.2 (2.0)	0.28
Being able to eat a lot (points), mean (SD)	6.3 (2.2)	6.4 (2.1)	6.3 (2.3)	0.95
Wanting to eat something sweet (points), mean (SD)	5.7 (2.4)	5.5 (2.4)	6 (2.5)	0.60
Wanting to eat something salty (points), mean (SD)	5.5 (2.3)	5.4 (1.9)	5.7 (2.8)	0.71
Wanting to eat something spicy (points), mean (SD)	5.2 (2.8)	4.6 (2.6)	5.8 (2.8)	0.26
Wanting to eat something oily (points), mean (SD)	6.0 (2.6)	5.3 (2.6)	6.7 (2.4)	0.17
Daily caloric intake per kg body weight (kcal/kg), mean (SD)	33.4 (14.6)	39.6 (14.8)	26.8 (11.4)	0.02*
Daily protein intake per kg body weight (g/kg), mean (SD)	1.3 (0.5)	1.4 (0.4)	1.1 (0.4)	0.08
Quality of life by EQ-5D				
Problem on mobility, n (%)				0.31
No problem	11 (40.7)	7 (50.0)	4 (30.8)	
Some problems	16 (59.3)	7 (50.0)	9 (69.2)	
Confined to bed	0	0	0	
Problem on self-care, n (%)				0.56
No problem	22 (81.5)	12 (85.7)	10 (76.9)	
Some problems	5 (18.5)	2 (14.3)	3 (23.1)	
Unable to do	0	0	0	
Problem with usual activity, n (%)				0.58
No problem	20 (74.1)	11 (78.6)	9 (69.2)	
Some problems	7 (25.9)	3 (21.4)	4 (30.8)	
Unable to perform	0	0	0	
Problem with pain or discomfort, n (%)				0.51
No pain	6 (22.2)	4 (28.6)	2 (15.4)	
Moderate pain or discomfort	20 (74.1)	9 (64.3)	11 (84.6)	
Extremely pain or discomfort	1 (3.7)	1 (7.1)	0	
Problem with anxiety or depression, n (%)				0.12
Not anxious or depressed	19 (70.4)	12 (85.7)	7 (53.9)	
Moderately anxious or depressed	6 (22.2)	1 (7.1)	5 (38.5)	
Extremely anxious or depressed	2 (7.4)	1 (7.1)	1 (7.6)	
VAS of health (points), mean (SD)	66.3 (14.7)	67.9 (12.2)	64.6 (17.5)	0.58

*SD* Standard deviation, *WHO* World Health Organization, *mRSS* Modified Rodnan skin score, *Hb* Hemoglobin, *ESR* Erythrocyte sedimentation rate, *CRP* C-reactive protein, *TGFβ* Transforming growth factor β, *IQR* Interquartile range, *VAS* Visual analogue scale

^a^Comparison between treatment and placebo group

*Statistically significant

### Outcomes comparison between the treatment and the placebo group

Thirteen patients in each group were included in the analysis. The details of these findings are presented in Table 2.

**Table 2:** Outcomes between treatment and placebo

Clinical characteristics at week 4 after treatment	Treatment group *N* = 13	Placebo group *N* = 13	*p*-value
Mean difference of body weight (kg), mean (SD)	0.15 (1.4)	−0.3 (1.2)	0.36
Increase in body weight, *n* (%)	5 (38.5)	4 (30.8)	> 0.99
Mean difference of body mass index (kg/m^2^), mean (SD)	0.05 (0.6)	−0.1 (0.5)	0.41
Increase in body mass index, *n* (%)	5 (38.5)	4 (30.8)	> 0.99
Mean difference of Hb (g/dL), mean (SD)	−0.2 (0.9)	0.2 (0.7)	0.25
Increase in Hb levels, *n* (%)	8 (61.5)	7 (53.9)	0.69
Mean difference of serum albumin (g/dL), mean (SD)	0.03 (0.16)	0.04 (0.20)	0.55
Increase in serum albumin, *n* (%)	7 (54.9)	4 (30.8)	0.23
Mean difference of ESR (mm/h), mean (SD)	2.4 (18.9)	6.6 (20.9)	0.64
Decrease in ESR levels, *n* (%)	5 of 10 (50.0)	3 of 10 (30.0)	0.65
Mean difference of CRP (mg/dL), median (IQR)	−0.4 (7.9)	3.4 (8.1)	0.74
Decrease in CRP levels, *n* (%)	7 of 12 (58.3)	5 (38.5)	0.32
Mean difference of transferrin levels (g/L), mean (SD)	0.04 (0.18)	−0.02 (0.18)	0.43
Increase in transferrin levels, *n* (%)	7 (53.8)	7 (53.8)	> 0.99
Mean difference of TGFβ (pg/mL), mean (SD)	0.4 (2.6)	−0.3 (1.7)	0.37
Decrease in TGFβ levels, *n* (%)	5 (38.5)	7 (53.9)	0.43
Mean difference of VAS for self-reported			
Hunger (points), mean (SD)	1.2 (1.1)	0.5 (2.7)	0.40
Satisfaction in eating (points), mean (SD)	1.1 (2.1)	0.7 (2.7)	0.69
Feeling of fullness (points), mean (SD)	−0.2 (2.5)	−0.6 (1.9)	0.66
Being able to eat a lot (points), mean (SD)	1.4 (2.4)	0.5 (3.0)	0.39
Wanting to eat something sweet (points), mean (SD)	−0.5 (2.5)	0.3 (2.2)	0.41
Wanting to eat something salty (points), mean (SD)	−0.9 (2.3)	−0.4 (2.5)	0.57
Wanting to eat something spicy (points), mean (SD)	0.6 (2.8)	−0.9 (2.2)	0.13
Wanting to eat something oily (points), mean (SD)	0.5 (2.7)	−0.2 (1.0)	0.40
Mean difference of daily caloric intake per kg body weight (kcal/kg), mean (SD)	6.3 (10.2)	6.2 (18.8)	0.99
Increase daily caloric intake per kg body weight, *n* (%)	9 (69.2)	9 (69.2)	> 0.99
Mean difference of daily protein intake per kg body weight (g/kg), mean (SD)	0.2 (0.5)	0.2 (0.7)	0.87
Increase daily protein intake per kg body weight, *n* (%)	9 (69.2)	9 (69.2)	> 0.99
Quality of life by EQ-5D, *n* (%)			
Improved mobility	12 (92.3)	12 (92.3)	> 0.99
Improved self-care	12 (92.3)	11 (84.6)	0.54
Improved usual activities	12 (92.3)	12 (92.3)	> 0.99
Improved pain or discomfort	12 (92.3)	13 (100.0)	> 0.99
Improved anxiety or depression	10 (76.9)	11 (84.6)	> 0.99
Mean difference of VAS of health (points), mean (SD)	5.8 (14.6)	−0.6 (19.2)	0.35

*SD* Standard deviation, *WHO *World Health Organization, *Hb H*emoglobin, *ESR* Erythrocyte sedimentation rate, *CRP *c-reactive protein, *TGFβ *Transforming growth factor β, *IQR *Interquartile range, *VAS* Visual analogue scale

### Changing of appetite

The treatment group had higher mean differences in VAS scores for hunger, satisfaction with eating, being able to eat a lot, wanting to eat something spicy, and wanting to eat something oily, but had lower mean differences in VAS for feeling fullness than the placebo group. However, the differences were not statistically significant. The treatment group also had lower mean differences in VAS for wanting to eat something sweet and salty than the placebo group, but the difference was not statistically significant.

### Changing body weight and food intake quantity

The treatment group showed an increase in body weight and BMI after treatment, whereas both parameters decreased in the placebo group. However, the differences in mean body weight and BMI between the groups were not statistically significant. Additionally, there were no significant differences in the mean changes in daily caloric intake per kg body weight or daily protein intake per kg body weight.

### Changing transferrin levels, cytokines levels, and inflammatory markers

The mean transferrin and hemoglobin (Hb) levels were not significantly different between the groups. ESR and CRP were reduced more in the treatment group than in the placebo group after treatment, but these differences were not statistically significant, nor were there significant differences in TGF-β levels.

### Changing of quality of life evaluated by EQ-5D

The comparison of quality of life evaluated by EQ-5D between the treatment and placebo groups showed no significant differences. However, the mean difference in the VAS for health tended to be higher in the treatment group than in the placebo group (5.8 vs. −0.6, *p* = 0.35).

### Outcomes comparison between baseline and 4 weeks after treatment

#### Changing of appetite

The mean differences in the VAS scores for hunger before and after treatment were significantly higher in the treatment group (5.9 VS. 7.2, *p* < 0.001) but not in the placebo group (5.5 VS. 6.1, *p* = 0.49). The mean differences in VAS scores for satisfaction with eating and being able to eat a lot trended to increase after treatment in the treatment group, but the difference was not statistically significant (*p* = 0.09 and *p* = 0.06, respectively). The VAS scores for feelings of fullness and wanting to eat something salty were reduced after treatment compared with baseline in both groups, but the difference was not statistically significant (Table 3).

**Table 3:** Clinical outcomes before and 4 weeks after treatment with treatment or placebo

Data	Treatment group *N* = 13		*p*-value	Placebo group *N* = 13		*p*-value
	Before treatment	After treatment		Before treatment	After treatment	
Body weight (kg), mean (SD)	44.5 (5.5)	44.7 (6.3)	0.71	46.2 (6.4)	46.1 (6.9)	0.34
Body mass index (kg/m^2^), mean (SD)	18.1 (2.1)	18.1 (2.4)	0.74	18.1 (1.6)	17.9 (1.5)	0.37
Laboratory tests						
Hb (g/dL), mean (SD)	12.3 (2.0)	12.0 (1.8)	0.32	11.8 (1.4)	11.9 (1.1)	0.44
Serum albumin (g/dL), mean (SD)	4.1 (0.4)	4.2 (0.4)	0.45	3.8 (0.4)	3.8 (0.5)	> 0.99
ESR (mm/h), mean (SD)	67.7 (27.2)	61.9 (20.8)	0.70	74.0 (34.4)	78.5 (35.1)	0.34
CRP (mg/dL), median (IQR)	3.1 (1.0–8.4.0.4)	1.6 (1.0–4.5.0.5)	0.34	2.4 (1.8–5.6)	2.6 (2.1–9.8)	0.34
Transferrin levels (g/L), mean (SD)	2.4 (0.3)	2.4 (0.4)	0.45	1.9 (0.4)	1.9 (0.4)	0.73
TGFβ (pg/mL), mean (SD)	5.8 (2.5)	6.2 (2.9)	0.57	6.1 (2.3)	5.7 (2.5)	0.45
VAS for self-reported						
Hunger (points), mean (SD)	5.9 (1.0)	7.2 (1.7)	< 0.001*	5.5 (3.0)	6.1 (1.9)	0.49
Satisfaction in eating (points), mean (SD)	5.0 (1.9)	6.1 (2.7)	0.09	5.5 (1.9)	6.2 (2.3)	0.37
Feeling of fullness (points), mean (SD)	7.9 (1.6)	7.7 (1.7)	0.74	7.2 (2.0)	6.6 (1.6)	0.26
Being able to eat a lot (points), mean (SD)	6.5 (2.1)	7.9 (1.4)	0.06	6.3 (2.3)	6.8 (2.0)	0.59
Wanting to eat something sweet (points), mean (SD)	5.5 (2.5)	5.1 (3.0)	0.51	6.0 (2.5)	6.3 (2/2)	0.62
Wanting to eat something salty (points), mean (SD)	5.4 (1.9)	4.5 (2.2)	0.17	5.7 (2.8)	4.9 (1.9)	0.58
Wanting to eat something spicy (points), mean (SD)	4.9 (2.5)	5.5 (3.2)	0.44	5.8 (2.8)	4.9 (2.8)	0.16
Wanting to eat something oily (points), mean (SD)	5.6 (2.4)	6.2 (2.1)	0.49	6.7 (2.4)	6.5 (2.6)	0.58
Daily calories intake per kg body weight (kcal/kg), mean (SD)	38.9 (15.1)	45.2 (15.6)	0.046*	26.8 (11.4)	33.0 (16.9)	0.25
Daily protein intake per kg body weight (g/kg), mean (SD)	1.4 (0.8)	1.6 (0.6)	0.16	1.1 (0.4)	1.3 (0.5)	0.41
VAS of health (points), mean (SD)	69.2 (11.6)	74.9 (13.8)	0.18	64.6 (17.5)	64.0 (13.9)	0.91

*SD* Standard deviation, *WHO *World Health Organization, *Hb* Hemoglobin, *ESR *Erythrocyte sedimentation rate, *CRP *C-reactive protein, *TGFβ *Transforming growth factor β, *IQR *Interquartile range, *VAS *Visual analogue scale

*Statistically significant

### Changing body weight and food intake quantity

The mean body weight and BMI were comparable between baseline and post-treatment in the treatment group, but both decreased in the placebo group, although these changes were not statistically significant. Daily calorie intake per kg body weight significantly increased in the treatment group after treatment, with respective means of 45.2 kcal/kg at follow-up and 38.9 kcal/kg at baseline (*p* = 0.046). Daily calorie intake per kg of body weight also increased in the placebo group after treatment, but this change was not statistically significant. Daily protein intake per kg of body weight increased in both groups compared to baseline, but the change was not statistically significant.

### Changing transferrin levels, cytokines levels, and inflammatory markers

The mean transferrin levels in both the treatment and placebo groups were comparable between baseline and post-treatment (*p* = 0.45 and 0.73, respectively), as were serum albumin levels (*p* = 0.45 and > 0.99, respectively). The mean TGFβ levels did not differ between the treatment and placebo groups (*p* = 0.57 for the treatment group and 0.45 for the placebo group). The mean ESR and CRP levels decreased after treatment compared to baseline in the treatment group, whereas both increased after treatment in the placebo group. However, these changes were not statistically significant.

### Changing of quality of life evaluated by EQ-5D

The mean VAS score for health increased after treatment in the treatment group compared to baseline, but the difference was not statistically significant (74.9 vs. 69.2, *p* = 0.18). In contrast, the VAS score for health remained comparable between baseline and post-treatment in the placebo group (64.0 vs. 64.6, *p* = 0.91).

The comparison of outcomes between baseline and after treatment in both the treatment and placebo groups is presented in Table 3.

### Adverse events

Adverse events were reported in 10 patients in the treatment group and 7 in the placebo group. One patient in the treatment group developed thirst and polydipsia after taking a single drop of cannabis oil, which resulted in severe hyponatremia and drowsiness. She was hospitalized to correct her sodium levels and withdrew from the study. Additionally, two patients in the treatment group had somnolence, and two reported dizziness. The other adverse events are summarized in Table 4. The incidence of adverse events did not significantly differ between the treatment and placebo groups.

**Table 4:** Adverse events

Adverse events	Treatment group *N* = 14	Placebo group *N* = 13
Aphthous ulcer	0	1
Bloating	1	0
Bronchitis	1	1
Diarrhea	0	1
Digital ulcer	0	1
Dizziness	2	0
Dry eyes	1	0
Palpitation	1	1
Fatigue	0	1
Hypertension	0	1
Puffy face	1	0
Pyuria	1	0
Somnolence	2	0

## Discussion

This randomized placebo-controlled trial evaluated the effects of cannabinoids in a 1:1 ratio of THC to CBD on appetite, body weight, inflammatory markers, and quality of life in patients with SSc. Of the 27 participants, most were female and had the dcSSc subset. The baseline clinical characteristics were generally balanced between the treatment and placebo groups, except for higher transferrin levels and daily calorie intake per kilogram of body weight in the treatment group. This imbalance may have influenced the outcomes.

The treatment group demonstrated trends toward improved appetite, reflected in higher mean differences in VAS scores for hunger, satisfaction with eating, and ability to eat more compared to the placebo group. These findings align with previous studies suggesting cannabinoids may stimulate appetite by modulating the endocannabinoid system (Marzo and Matias 2005; Camilleri and Zheng 2023; Cohen and Neuman 2020). Specifically, THC is known to mediate appetite through activation of CB1 receptors. In addition, endocannabinoids facilitate mesolimbic dopamine signaling, enhance appetite, and promote eating initiation and food seeking (Kirkham 2009). Although cannabinoids in THC-based formulations demonstrated favorable outcomes on appetite in 11 patients with advanced cancer compared to placebo (Brisbois et al. 2011), many studies on cannabinoids for appetite stimulation in patients with cancer reported similar findings to ours, showing no significant differences in VAS scores for appetite between the treatment and placebo groups (Cannabis-In-Cachexia-Study-Group et al. 2006; Côté et al. 2016; Turcott et al. 2018). One study also reported a significant improvement in appetite compared to baseline, consistent with our findings (Turcott et al. 2018). Other appetite-related parameters, such as feelings of fullness and wanting to eat salty foods, showed no significant changes between or within groups, but the VAS seemed to be lower in the treatment group than in the placebo group. This suggests that cannabinoids may not significantly affect the mechanism underlying the feeling of fullness.

Our analysis suggests that cannabinoids may enhance sweet taste. A previous study in an animal model showed that endocannabinoids can increase sweet taste perception by stimulating CB1 receptors in taste cells, counteracting the inhibitory effects of leptin on sweet taste (Yoshida et al. 2010). The results corroborate our observations that patients in the treatment group exhibited an elevation in the VAS score for craving sweet foods following cannabis consumption, relative to baseline measurements. Cannabinoids also trended to increase the VAS score for wanting to eat spicy foods after taking cannabinoids when compared to baseline. No mechanism has been previously shown to corroborate these findings, as spiciness may not constitute a conventional dietary element in daily life, as it does within the Thai population. However, these findings may guide therapeutic approaches for managing appetite and energy homeostasis in the clinical setting. The mechanisms of the spicy taste of cannabinoids require further investigation.

In contrast to the decrease observed in the placebo group, the treatment group showed a slight increase in body weight and BMI mass index, although these differences were not statistically significant. These findings are consistent with evidence indicating cannabinoids can enhance caloric intake in conditions characterized by anorexia or wasting (Haney et al. 2007) but no significant weight gain among patients with cancer (Cannabis-In-Cachexia-Study-Group et al. 2006; Côté et al. 2016; Turcott et al. 2018). In contrast, a previous observational prospective study in 282 malnourished older participants in nursing homes reported that after taking 134 kcal/d more, the mean weight gain was 2.3 kg at 12 weeks (Malafarina et al. 2021). That means body weight might increase at least 0.7 kg in 4 weeks if taking an additional 134 kcal/d. Although our patients had significant increases in daily calorie intake, the rate of weight gain was lower than expected. These results might be explained by other factors affecting BW, such as gastrointestinal (GI) involvement in SSc, which may cause malabsorption or maldigestion. The lack of significant weight gain, despite increased caloric intake in both groups, may be explained by several factors. First, the presence of GI involvement in approximately 20% of our cohort could have led to malabsorption, thereby blunting the impact of increased food consumption on body weight. Second, the short follow-up duration was a key limitation. As weight gain is often a gradual process, our four-week study period may have been insufficient to detect meaningful changes. Future trials should therefore incorporate longer treatment and follow-up periods to more accurately assess this outcome. Finally, given the imbalances in some baseline characteristics, the results must be interpreted with caution as these differences may have confounded the outcomes. To date, the 2016 European Society for Parenteral and Enteral Nutrition has not graded the use of cannabinoids to improve appetite in cancer patients, rating it as having a low level of evidence, with no recommendation provided for other patient groups (Johnson et al. 2021). Further well-designed studies with larger populations are needed.

Markers of inflammation, including ESR and CRP, decreased in the treatment group but increased in the placebo group after treatment. Cannabinoids have been reported to reduce inflammation in inflammatory bowel disease (IBD), as evidenced by improvements in the Crohn’s Disease Activity Index compared to placebo (Naftali et al. 2013). Another study demonstrated that cannabinoids improved quality of life and appetite in IBD patients compared to placebo, although CRP levels did not significantly change (Naftali et al. 2021). Henshaw et al.(2021) conducted a systematic review of 26 studies examining the anti-inflammatory effects of cannabinoids. Two studies specifically evaluated the combined effects of THC and CBD, revealing that this formulation reduced tumor necrotic factor-α (TNF-α) levels in vivo. The anti-inflammatory effects of cannabinoids vary by dose (Henshaw et al. 2021) and have also been reported to decrease levels of interleukin-1 (IL-1), interleukin-6 (IL-6), and interleukin-12 (IL-12) (Klein 2005). As IL-1, IL-6, IL-12, and TNF-α are not the primary cytokines involved in SSc; therefore, we did not evaluate their levels in our study. While the reductions in ESR and CRP in the treatment group were not statistically significant, they suggest the potential anti-inflammatory effects of cannabinoids. These findings highlight the need for more extensive trials incorporating pro- and anti-inflammatory cytokines to confirm these effects and to better understand their role in SSc management.

Cannabinoids may not directly influence TGF-β, a key cytokine in fibrosis. Our findings showed no significant differences in TGF-β levels between the treatment and placebo groups and notable changes after treatment. Gonzalez et al. reported antifibrotic effects in SSc-induced mice treated with ajulemic acid, a synthetic cannabinoid with a higher binding affinity for CB2 than for CB1 (Gonzalez et al. 2012). Among cannabinoids, CBD has potential immunomodulatory and anti-inflammatory effects. At the same time, THC is known for its effects on appetite stimulation, antiemesis, and analgesia (Zurier and Burstein 2016). The antifibrotic effect of ajulemic acid may be explained by its activation of peroxisome proliferator-activated receptor-γ (PPARγ), which subsequently disrupts the signaling of TGF-β, a major fibrogenic cytokine (Gonzalez et al. 2012). However, the exact mechanism of both anti-inflammatory and antifibrotics of cannabinoids, particularly those with predominant activity on CB2, remains poorly understood.

Improvements in quality of life, measured by the EQ-5D health VAS, were observed in the treatment group post-treatment, although the change was not statistically significant. These findings are consistent with studies suggesting cannabinoids may have positive effects on health perception and overall well-being in chronic disease patients (Whiting et al. 2015). 

While cannabinoids have many potential benefits, safety and tolerability data are essential for balanced interpretation. Acute toxicity is a known risk and depends on the dose, patient tolerance, and route of administration. Cannabis can also influence brain functions, including memory and cognition, and increases the risk of psychosis in individuals with prolonged use (Vadivelu et al. 2018). The symptoms of central nervous system (CNS) toxicity can include euphoria, panic, agitation, mood alterations, perceptual changes, loss of social inhibition, muscle incoordination, myoclonic jerking, ataxia, slurred speech, and suicidal ideation. Furthermore, prolonged, high-dose cannabis use can lead to cannabinoid hyperemesis syndrome, which causes cyclic hyperemesis (Allen et al. 2004) and can ultimately result in electrolyte disturbances and impaired kidney function (Vadivelu et al. 2018). The criticality of this was highlighted when one patient in our study experienced a serious adverse event after a single drop of cannabis oil, necessitating immediate withdrawal from treatment and emergency care. This incident validates our study’s rigorous safety protocol, which included close clinical and laboratory monitoring for emergent toxicities, even after the first dose. Our management strategy for such events ranged from dose reduction to immediate treatment discontinuation to always ensure patient safety.

None of the patients developed neuropsychological complications related to the psychotropic effects of cannabinoids, particularly from THC, and all tolerated the daily dose of 2.9 mg THC and 3.2 mg CBD, except for one case with a serious adverse reaction after a single drop of cannabinoid. This may be attributed to the low doses of cannabinoids used in our study. Brisbois et al. (2011) found that THC in a dose of 2.5 mg twice daily had a more significant effect on food taste, quality of life, and sleep quality than placebo treatment in cancer patients. Another study revealed that the effect of increasing appetite by THC at a dose of 2.5 mg twice daily was not more significant than that of megestrol 800 mg daily, and there was no additional benefit when THC was combined with megestrol (Vadivelu et al. 2018). In our study, the doses of THC (1.5 mg twice daily) and CBD (1.6 mg twice daily) were lower than those reported in previous studies but still led to improved appetite in SSc patients. Although the effects of cannabinoids can vary with dosage, we cannot conclude whether a higher dose results in more significant improvements in appetite, quality of life, transferrin levels, or reduction in inflammatory markers and cytokines.

The study drug was administered by transmucosal route (sublingual drop), which is claimed to have good absorption and high bioavailability route when compared to oral ingestion because of direct uptake of the drug by the highly vascularized oral mucosa and does not pass the digestive system, so the drug is not interfering with absorption by gastric acid and does not metabolize via the liver (Stella et al. 2021). Our patients administered cannabis sublingually at a reduced dosage and still achieved efficacy. However, the sublingual route may be more difficult to administer than oral ingestion because absorption is influenced by eating, drinking, or smoking. Patients should be advised to take the drug while fasting. To overcome the limitation, the production of cannabinoids with new technology, such as nanotechnology, may help improve the bioavailability of oral route administration and increase physical stability when compared to conventional formulations (Stella et al. 2021). 

The limitations of this study include: (a) a small sample size, which likely left the study underpowered to detect significant differences and increased the risk of a Type II error. Consequently, our findings have limited generalizability and require validation in larger, multicenter randomized controlled trials; (b) baseline imbalances in transferrin levels and calorie intake, which may have introduced bias, although we adjusted for calorie intake per kilogram daily; c) a short follow-up period, which may have limited the detection of changes in parameters, requiring more time to show significant effects, such as serum albumin and body composition; (d) body composition was not included in our study for the reasons mentioned; and (e) the use of an unconventional dose in daily practice due to limitations in extracting plant-derived exogenous cannabinoids. The strengths of this study include being the first to investigate the efficacy of cannabinoids on appetite in SSc patients and to analyze the quality of life, key cytokine levels, inflammatory markers, and daily caloric intake. Future studies should involve larger cohorts and stratify patients according to disease subtype or severity to better understand the heterogeneity of the responses.

## Conclusion

Cannabis oil demonstrated a trend toward improving appetite, satisfaction with eating, body weight, daily calorie intake, and quality of life in SSc patients with anorexia or malnutrition. While most outcome measures did not reach statistical significance, the treatment group showed a significant increase in hunger VAS scores. Additionally, reductions in inflammatory markers were observed, though not significantly different from placebo. The treatment was generally well tolerated. Further studies with larger sample sizes and longer follow-up periods are needed to confirm the potential benefits of cannabinoids in SSc-related anorexia and inflammation.

## Supplementary Information

Supplementary Material 1. CONSORT checklists.

## Data Availability

The datasets used and/or analysed during the current study are available from the corresponding author on reasonable request.

## Abbreviations

ACR: American College of Rheumatology
BMD: Body mass index
BW: Body weight
CBD: Cannabidiol
CB1: Cannabinoid receptor 1
CB2: Cannabinoid receptor 2
CRP: C-reactive protein
dcSSc: Diffuse cutaneous systemic sclerosis
EQ-5D: EuroQol-5 Dimension
ESR: Erythrocyte sedimentation rate
EULAR: European Alliance of Associations for Rheumatology
GI: Gastrointestinal
Hb: Hemoglobin
IBD: Inflammatory bowel disease
IL-1: Interleukin-1
IL-6: Interleukin-6
IL-12: Interleukin-12
lcSSc: Limited cutaneous systemic sclerosis
NSAIDs: Non-steroidal anti-inflammatory agents
PPARγ: Peroxisome proliferator-activated receptor-γ
SSc: Systemic sclerosis
TGF-β: Transforming growth factor-β
TNF-α: Tumor necrotic factor-α
QoL: Quality of life
SD: Standard deviation
THC: Tetrahydrocannabinol
VAS: Visual analogue scale
